# Assessment of low-dose paranasal sinus CT imaging using a new deep learning image reconstruction technique in children compared to adaptive statistical iterative reconstruction V (ASiR-V)

**DOI:** 10.1186/s12880-022-00834-1

**Published:** 2022-06-03

**Authors:** Yang Li, Xia Liu, Xun-hui Zhuang, Ming-jun Wang, Xiu-feng Song

**Affiliations:** 1 Department of Radiology, Qingdao University Affiliated Women and Children’s Hospital, 6 Tongfu Road, Qingdao, Shandong 266034 China; 2 Department of Abdominal Ultrasound, The Affiliated Hospital of Qingdao University, Qingdao, 266003 China; 3 GE Healthcare (Shanghai) Co., Ltd., Shanghai, 201203 China

**Keywords:** Artificial intelligence, Deep learning, Iterative reconstruction, Paranasal sinuses, Radiation reduction, Children

## Abstract

**Purpose:**

To compare the effects of deep learning image reconstruction (DLIR) and adaptive statistical iterative reconstruction V (ASiR-V) on image quality in low-dose computed tomography (CT) of paranasal sinuses in children.

**Methods:**

Low-dose CT scans of the paranasal sinuses in 25 pediatric patients were retrospectively evaluated. The raw data were reconstructed with three levels of DLIR (high, H; medium, M; and low, L), filtered back projection (FBP), and ASiR-V (30% and 50%). Image noise was measured in both soft tissue and bone windows, and the signal-to-noise ratios (SNRs) and contrast-to-noise ratios (CNRs) of the images were calculated. Subjective image quality at the ethmoid sinus and nasal cavity levels of the six groups of reconstructed images was assessed by two doctors using a five-point Likert scale in a double-blind manner.

**Results:**

The patients’ mean dose-length product and effective dose were 36.65 ± 2.44 mGy·cm and 0.17 ± 0.03 mSv, respectively. (1) Objective evaluation: 1. Soft tissue window: The difference among groups in each parameter was significant (P < 0.05). Pairwise comparisons showed that the H group’ s parameters were significantly better (P < 0.05) than those of the 50% post-ASiR-V group. 2. Bone window: No significant between-group differences were found in the noise of the petrous portion of the temporal bone or its SNR or in the noise of the pterygoid processes of the sphenoids or their SNRs (P > 0.05). Significant differences were observed in the background noise and CNR (P < 0.05). As the DLIR intensity increased, image noise decreased and the CNR improved. The H group exhibited the best image quality. (2) Subjective evaluation: Scores for images of the ethmoid sinuses were not significantly different among groups (P > 0.05). Scores for images of the nasal cavity were significantly different among groups (P < 0.05) and were ranked in descending order as follows: H, M, L, 50% post-ASiR-V, 30% post-ASiR-V, and FBP.

**Conclusion:**

DLIR was superior to FBP and post-ASiR-V in low-dose CT scans of pediatric paranasal sinuses. At high intensity (H), DLIR provided the best reconstruction effects.

## Introduction

Due to the special physiological changes in the development of children’ s paranasal sinuses, they are prone to develop swollen mucous membranes and increased secretion, which can cause sinusitis. CT examination is intuitive and fast, and it is the preferred method for examining the paranasal sinuses of children. However, because the scan region for the sinus inevitably overlaps with the orbit, potential irradiation of the vitreous body is always a concern in paranasal sinus CT [1]. Therefore, it is important to investigate how to minimize the radiation dose while maintaining clinically acceptable image quality. Reducing the CT radiation dose in children has been a promising focus of research in recent years [2–4].

Filtered back projection (FBP) is the mainstream standard for CT image reconstruction, but FBP technology is limited by significant noise and artifacts [5, 6]. In addition, it does not provide excellent diagnostic images with low radiation doses. With the rapid advancement of iterative reconstruction (IR) technology in recent years, various CT suppliers have introduced advanced IR algorithms to reduce the radiation dose. Adaptive statistical iterative reconstruction V (ASiR-V) technology is a promising technology that has been researched in recent years and can provide high-quality diagnostic images with significantly reduced radiation doses [7, 8]. However, it is greatly limited because it is excessively smooth and unnatural. Deep learning image reconstruction (DLIR) is a novel image reconstruction method. This technology has been configured with high-dose FBP data sets and deep neural network (DNN) models to further reduce noise and suppress artifacts [9]. DLIR technology has not been in use long enough to obtain Food and Drug Administration certification and has not been promoted extensively, and no research on its application to CT scanning of the paranasal sinus in children has been reported. This study aims to compare the impacts of DLIR and ASiR-V on the image quality of low-dose CT scans of the nasal sinuses of children and to conduct an initial investigation of the application value of DLIR technology in CT scans of the paranasal sinus in children.

## Materials and methods

### General information

Twenty-five children (13 males and 12 females) with suspected paranasal sinusitis were imaged with low-dose paranasal sinus CT at our hospital between February and March 2020. The patients were aged 2–14 years, with a mean age of 6.84 ± 2.98 years. This study is based on raw data from our routine low-dose scans and did not involve changes in the scan parameters we use clinically. This study was therefore exempted from the requirement for written informed consent and approved by the institutional review board of our hospital.

### Instruments and methods

All patients underwent paranasal sinus CT with a 256-detector row scanner (Revolution CT; General Electric Healthcare, Waukesha, WI, USA). The scanning conditions were set as follows: 100 kVp, SmartmA 60–250 mA, pre-ASiR-V 70%, noise index 22, pitch 0.992:1, rotation time 1.00 s, slice thickness 1.25 mm, and interslice spacing 1.25 mm. Patients in the supine position underwent an axial scan, ranging from the upper jaw to the top of the frontal sinus, while the radiation-sensitive organs of the children, such as the gonads, were covered and protected. Children who did not cooperate were sedated with an oral dose or enema of chloral hydrate. The CT dose index (CTDI) and dose-length product (DLP) were recorded.

### Image postprocessing and analysis

The raw data were reconstructed using six groups of reconstructed images with three levels of DLIR (high, medium, and low), ASiR-V at 30% and 50%, and FBP (ASiR-V 0%), denoted H, M, L, AV50, AV30, and FBP, respectively. The slice thickness and interslice gap in reconstruction were both 0.625 mm. Reconstructed images were transmitted to an AW4.7 workstation for objective and subjective evaluations in the soft tissue window and bone window. The bone window data were acquired by setting the Edge 3 filter on the AW workstation. The width of the soft tissue window was 100 HU, with a window center of 45 HU, while the bone window width was 1100 HU, with a window center of 900 HU.

#### Objective evaluation

Regions of interest (ROIs) sized 10 mm2 were applied on three consecutive images at the same location to obtain the mean value for each reconstruction. CT values of the turbinate mucosa, infratemporal fossa fat, and nasopharyngeal air were measured on soft tissue window images. The standard deviation (SD) of the CT values was taken as noise, and the SD value for nasopharyngeal air was taken as the background noise to calculate the signal-to-noise ratio (SNR) and contrast-to-noise ratio (CNR) using the formulas SNR = CT value/SD value and CNR = (turbinate mucosa CT value—inferior temporal fossa fat CT value)/(SD value of the nasopharyngeal air), respectively. CT values of the petrous portion of the temporal bone, pterygoid processes of the sphenoids, and the air in the anterior maxillary sinus were measured on bone window images. The SD value of the CT values was taken as noise, and the SD value for the air in the anterior maxillary sinus was taken as the background noise to calculate the SNR and CNR values using the formulas SNR = CT value/SD value and CNR = (CT value of the petrous part of the temporal bone—CT value of the pterygoid processes of the sphenoids)/(SD value of the air in the anterior maxillary sinus), respectively.

#### Subjective scores

Subjective evaluations of six reconstructed images were conducted by two doctors using a five-point Likert scale in a double-blind manner. The quality of images of the ethmoid sinus and nasal cavity was evaluated. The scoring standard was as follows: one point (excellent) for images that had no obvious artifacts and had clear anatomical details and sharp edges, two points (good) for images that had slight artifacts but had clear anatomical details, three points (medium) for images with significant artifacts but with acceptable anatomical structures meeting the diagnostic needs, four points (poor) for images with strong artifacts and unclear details of local anatomical features with great influence on the diagnosis, and five points (no diagnostic value) for images with significant artifacts that were unsuitable for diagnosis.

### Radiation dose

The DLP was recorded to calculate the effective dose (ED) with the formula ED = DLP × K, where K is the conversion factor [10].

### Statistical analysis

Objective data were tested by Welch ANOVA, and the Dunnett T3 test was used for comparisons between two groups. The interrater reliability between the two physicians in scoring was verified by the kappa test, and the subjective scores were tested by the Kruskal–Wallis test. Statistical analysis was performed with SPSS 21 software, and statistical significance was accepted at P < 0.05.

## Results

The mean volume CTDI (CTDIvol) was 2.35 mGy, the mean DLP was 36.65 ± 2.44 mGy·cm (range 31.06–41.35), and the mean ED was 0.13 ± 0.02 mSv (range 0.10–0.20). The radiation dose was significantly lower than the doses recommended by the European guidelines on quality criteria for CT (360 mGy·cm).

### Quantitative image assessment

(1) Soft tissue window: All measured image quality indicators were significantly different between the six reconstructed images in the soft tissue window (P < 0.05). As the DLIR and ASiR-V intensity increased, the noise decreased, and the SNR and CNR increased. Parameters in the H group (the best group among the DLIR groups) were significantly better (P < 0.05) than those in the 50% post-ASiR-V group (the best among the post-ASiR-V groups) (P < 0.05). (2) Bone window: The noise and SNRs of the petrous portion of the temporal bone and the pterygoid processes of the sphenoids were not significantly different (P > 0.05). Significant differences were observed in the images’ background noise and CNRs (P < 0.05). As the DLIR intensity increased, image noise decreased, and the CNR improved (Tables 1, 2). The H group exhibited the best image quality in the bone window.

**Table 1:** Objective evaluation of images with different reconstruction algorithms

Reconstruction algorithm	Inferior meatus level						Temporal bone level					
	Inferior turbinate mucosa Noise	Infratemporal fossa Noise	Image Noise	Inferior turbinate mucosa SNR	Infratemporal fossa SNR	CNR	Petrosal bone Noise	Pterygoid process Noise	Image Noise	Petrosal bone SNR	Pterygoid process SNR	CNR
DLIR-high	10.14 ± 2.45	11.56 ± 3.42	8.12 ± 2.45	4.26 ± 1.91	9.73 ± 3.80	18.68 ± 5.28	140.98 ± 40.21	76.44 ± 20.66	9.94 ± 1.29	14.05 ± 4.33	0.52 ± 0.24	189.66 ± 23.15
DLIR-medium	11.77 ± 2.45	13.77 ± 3.45	9.93 ± 2.7	3.58 ± 1.28	7.14 ± 3.92	14.34 ± 6.83	143.60 ± 50.94	79.34 ± 18.93	14.23 ± 1.56	14.63 ± 6.01	0.49 ± 0.33	132.58 ± 18.56
DLIR-low	14.38 ± 6.93	15.24 ± 3.10	12.98 ± 4.43	2.98 ± 1.12	7.02 ± 2.59	11.74 ± 4.21	137.57 ± 36.66	81.41 ± 16.01	16.92 ± 2.36	14.53 ± 4.74	0.49 ± 0.27	113.30 ± 18.58
AsirV-50%	13.79 ± 2.59	15.86 ± 3.59	14.35 ± 3.08	2.86 ± 0.90	6.74 ± 2.16	10.18 ± 2.65	150.32 ± 37.54	81.24 ± 20.01	21.35 ± 2.30	13.09 ± 4.04	0.51 ± 0.30	88.49 ± 10.62
AsirV-30%	16.17 ± 3.62	18.31 ± 4.34	15.64 ± 3.81	2.56 ± 0.92	5.83 ± 1.72	9.53 ± 2.68	163.56 ± 40.34	8432 ± 21.94	25.79 ± 2.60	11.76 ± 4.33	0.44 ± 0.25	73.80 ± 6.69
FBP	19.41 ± 5.15	19.97 ± 3.99	17.60 ± 3.26	2.16 ± 0.78	5.41 ± 1.96	8.30 ± 2.00	151.87 ± 40.25	83.96 ± 17.73	28.02 ± 2.08	12.93 ± 3.60	0.42 ± 0.18	66.83 ± 5.74
F	19.49	15.72	36.36	8.25	6.03	19.39	1.34	0.51	362.65	1.28	0.81	190.36
P	P < 0.05	P < 0.05	P < 0.05	P < 0.05	P < 0.05	P < 0.05	P > 0.05	P > 0.05	P < 0.05	P > 0.05	P > 0.05	P < 0.05

**Table 2:** Comparison of noise and CNR in different reconstruction methods (DLIR-high) and (AsirV-50%)

Reconstruction algorithm	Inferior meatus level						Temporal bone level					
	Inferior turbinate mucosa Noise	Infratemporal fossa Noise	Image Noise	Inferior turbinate mucosa SNR	Infratemporal fossa SNR	CNR	Petrosal bone Noise	Pterygoid process Noise	Image Noise	Petrosal bone SNR	Pterygoid process SNR	CNR
DLIR-high	10.14 ± 2.45	11.56 ± 3.42	8.12 ± 2.45	4.26 ± 1.91	9.73 ± 3.80	18.68 ± 5.28	140.98 ± 40.21	76.44 ± 20.66	9.94 ± 1.29	14.05 ± 4.33	0.52 ± 0.24	189.66 ± 23.15
AsirV-50%	13.79 ± 2.59	15.86 ± 3.59	14.35 ± 3.08	2.86 ± 0.90	6.74 ± 2.16	10.18 ± 2.65	150.32 ± 37.54	81.24 ± 20.01	21.35 ± 2.30	13.09 ± 4.04	0.51 ± 0.30	88.49 ± 10.62
P	P < 0.05	P < 0.05	P < 0.05	P < 0.05	P < 0.05	P < 0.05	P > 0.05	P > 0.05	P < 0.05	P > 0.05	P > 0.05	P < 0.05

### Qualitative image assessment

**Fig. 1:**
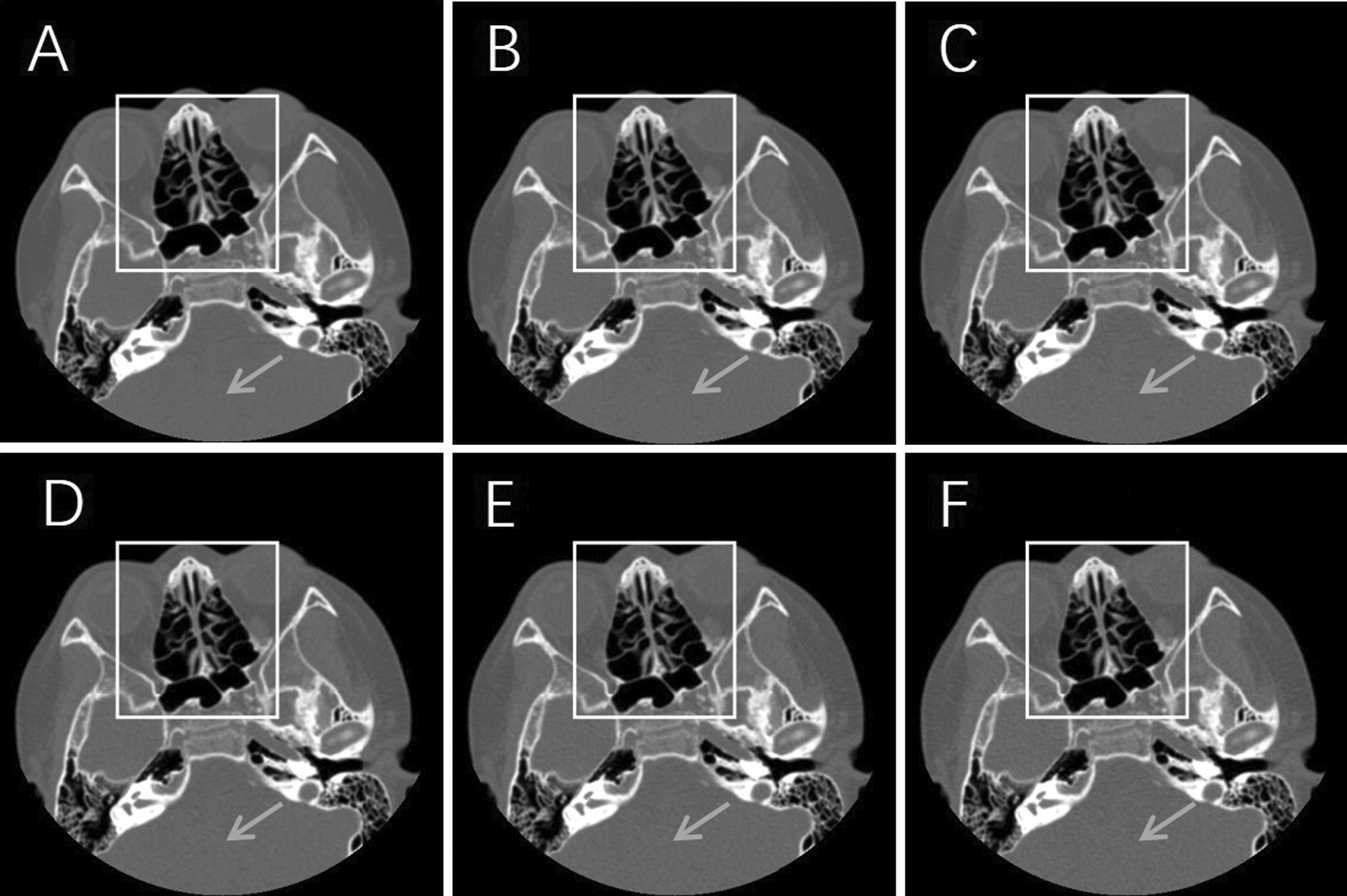
Axial CT bone window images from different reconstruction algorithms. **A** DLIR-high, **B** DLIR-medium, **C** DLIR-low, **D** AsirV-50%, **E** AsirV-30%, **F** FBP. The subjective image quality of the ethmoid sinuses (white box areas) were not significantly different among groups; only in some soft tissue areas the subjective perception of image graininess differed (gray arrows indicate areas). The images were excellent for mild sinusitis detection

**Fig. 2:**
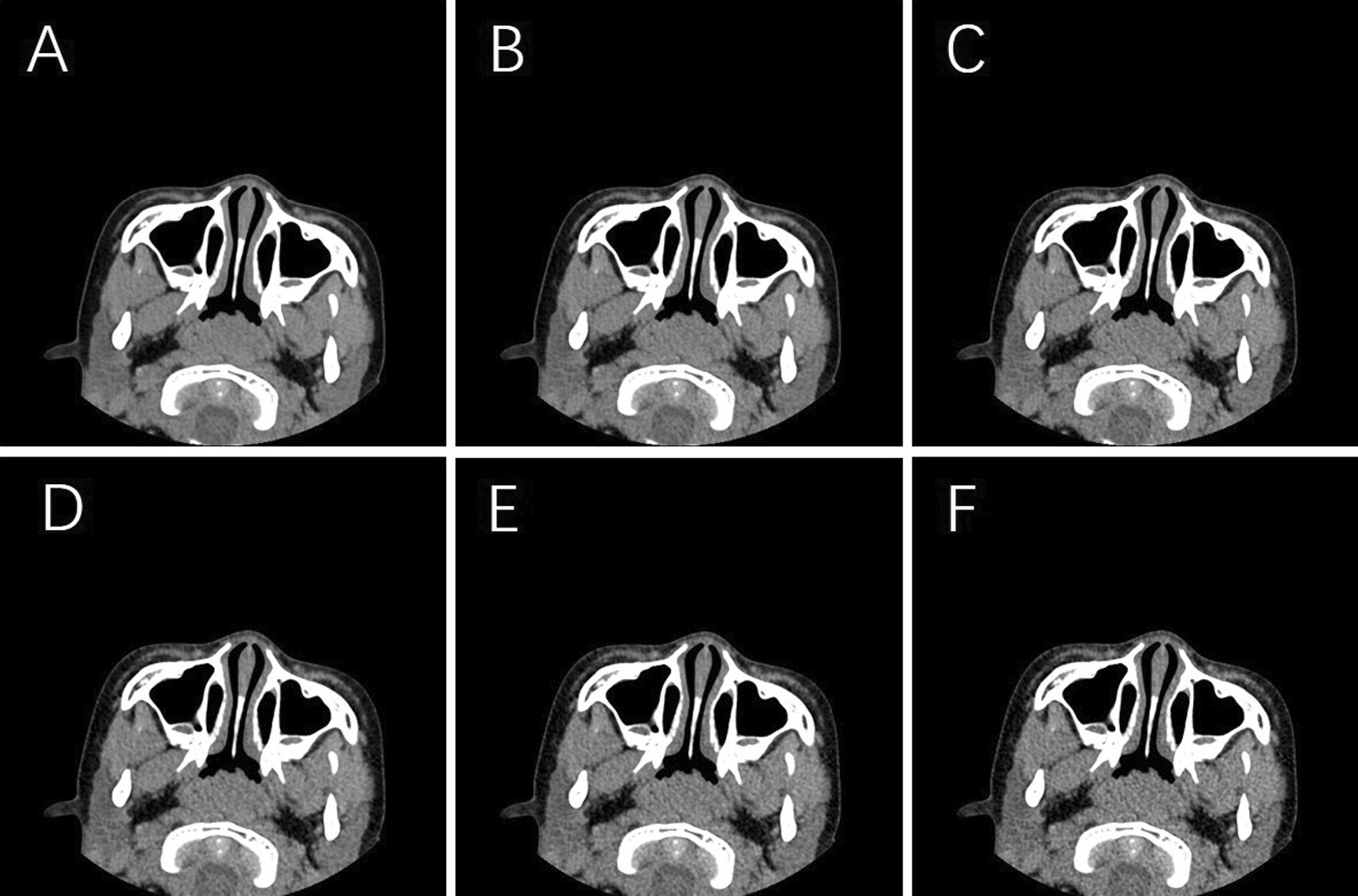
Axial CT soft tissue window images from different reconstruction algorithms. **A** DLIR-high, **B** DLIR-medium, **C** DLIR-low, **D** AsirV-50%, **E** AsirV-30%, **F** FBP. The subjective image quality, mainly evaluated based on image sharpness and graininess, was ranked, in descending order, as DLIR-high, DLIR-medium, DLIR-low, 50% post-ASiR-V, 30% post-ASiR-V, and FBP

(1) Two physicians with 8 and 15 years of working experience graded 125 images in five groups of 25 patients. The evaluation results of the two physicians showed moderate (kappa = 0.558) interrater reliability in the ethmoid sinuses and substantial (kappa = 0.676) interrater reliability in the nasal cavity, as verified by the kappa test. (2) The subjective image analysis results are presented in Table 3. Scores for images of the ethmoid sinuses were not significantly different among groups (P > 0.05) (Fig. 1). Scores for images of the nasal cavity were significantly different among groups (P < 0.05). The M (1.46 ± 0.58) and H (1.30 ± 0.61) groups had better subjective scores than the ASiR-V 50% group (1.82 ± 0.69). The scores for images were ranked in descending order as follows: H, M, L, 50% post-ASiR-V, 30% post-ASiR-V, and FBP (Fig. 2).

**Table 3:** Subjective image quality

Reconstruction algorithm	Ethmoidal cellules	Nasal cavity
DLIR-low	1.48 ± 0.50	1.96 ± 0.60
DLIR-medium	1.36 ± 0.48	1.46 ± 0.58
DLIR-high	1.30 ± 0.46	1.30 ± 0.61
FBP	1.60 ± 0.73	2.62 ± 0.88
AsirV-30%	1.52 ± 0.74	2.12 ± 0.59
AsirV-50%	1.28 ± 0.45	1.82 ± 0.69
χ^2^	8.81	88.70
P	P > 0.05	P < 0.05

## Discussion

During childhood, the nasal sinuses undergo pneumatization and development and contain rich mucosal vessels that easily lead to obstruction of the sinus ostium and sinusitis. Nasal sinus CT scans are useful for diagnosing sinusitis, but the scope of a nasal sinus CT scan will inevitably lead to irradiation of radiation-sensitive organs such as glands and the crystalline lens [11]. Therefore, dramatically reducing the radiation dosage in paranasal sinus CT scans, especially for pediatric patients, is critical to ensure clinical acceptance in the long run. In our study, we evaluated 25 pediatric patients who were at this particular physiological stage.

The paranasal sinuses comprise air-filled spaces in the skull and facial bones, and the sinus walls are thin. These sinuses have the advantage of high natural contrast. In addition, children have thinner tissue and lower bone density than do adults, which allows lower tube voltage and current during scanning. In this study, we applied 100 kVp, a reasonable tube voltage setting and low tube current to generate low-dose scan conditions. We also applied the fastest tube rotation time and pre-ASiR-V to further reduce the radiation dose. In our study, the radiation dose (DLP) was 36.65 ± 2.44 mGy·cm, an 89.82% reduction from that given in the European guidelines on quality criteria for pediatric paranasal sinus CT (360 mGy·cm). The dose in our study was at the same level as the mainstream low-dose scanning radiation dose, which can provide better scanning images.

In recent years, many methods have been proposed in various studies to realize low-dose scanning. Most of the early studies on low-dose scanning relied on FBP reconstruction, and the most common implementation methods were simple reduction of the tube voltage or tube current [15, 16]. Although such simple and direct methods can achieve a certain degree of radiation-dose reduction, the images are significantly limited by noise and artifacts. At present, there are multiple studies on low-dose CT scanning of the paranasal sinuses [12, 13], and even ultralow-dose scans have been reported [14]. We noticed that although some ultralow radiation dose scanning studies reduced the radiation dose to the lower limit, the image noise was high, and the CNR was also significantly reduced. This is acceptable for the diagnosis of sinusitis, but CT examination of maxillofacial trauma and temporal bone, which requires very high image sharpness, will affect the diagnosis of the disease. Therefore, a more advanced method is to use various IR technologies, which has led to a qualitative leap in the realization of low-dose scanning. Kong [17] used a 320-detector CT scanner combined with IR technology, reduced the radiation dose, and obtained good scanned images. Sun [18] used full model-based iterative reconstruction (MBIR) technology to perform an ultralow radiation dose CT examination and demonstrated that MBIR can provide equal or better image quality in paranasal sinus CT imaging of pediatric patients than can standard-dose CT with the ASiR algorithm. ASiR-V is currently one of the most advanced hybrid IR reconstruction technologies in commercial use. It is also the standard reconstruction algorithm that our Revolution CT uses. Therefore, we chose this technique as the standard control in our study. The ASiR-V algorithm innovatively presets weights before CT scanning and can significantly reduce the radiation dose and improve the quality of image reconstruction through more active noise reduction and improvement and application of object and physical models [7, 8]. Pre-ASiR-V can determine the radiation dose, and the ED value decreases as the weight increases, but after reaching the optimal weight range, the subjective scores of the images will decrease as the weight increases. Post-ASiR-V can improve the image quality, and as the weight ratio gradually increases, the image noise is reduced. However, after the optimal weight range is reached, the sharpness of the images gradually decreases, and the edges of the images gradually become blurred and distorted [19]. Therefore, our study used reasonable weight ratios, i.e., a pre-ASiR-V weight of 70% and post-ASiR-V weights of 30% and 50%.

Our study is the first to apply DLIR in low-dose pediatric paranasal sinus CT imaging. The image reconstruction techniques compared in our study were DLIR and ASiR-V. The DLIR algorithm (General Electric TrueFidelity) provided by the manufacturer used in our work is based on the deep convolutional neural network model. The characteristic of this deep learning algorithm is its use of high-quality, large-sample FBP data sets to train a DNN. During the training process, the DNN is used to analyze the data, a reconstruction function is synthesized and optimized through the learning process, and the inference engine is verified against a wide range of test data sets [9, 20, 21]. The DLIR algorithm that we used can provide reconstruction with high, medium, and low strengths to achieve different noise reduction effects. We compared the objective quality of the images in the commonly used soft tissue window and bone window. In soft tissue windows with high image quality requirements, by comparing the traditional ASiR-V algorithm and FBP, our study showed that high-level DLIR reconstruction yielded low-dose sinus CT scan images with significantly higher SNRs and CNRs and lower noise than those obtained with ASiR-V 50%. In bone window images, although no significant differences were observed in the noise and SNR of all measured features, the CNRs of DLIR images were significantly higher than those of ASiR-V and FBP images, and the background noise was significantly lower, which may be due to the greater natural contrast of bone window images and the smaller differences among various reconstruction algorithms. The subjective overall quality scores for DLIR images also showed the same trend, and the nasal cavity level DLIR was significantly better than the ASiR-V and FBP images. Similarly, with the objective quality of images in the bone window, the subjective scores of ethmoid cellules were not significantly different for this same reason. More advanced reconstruction algorithms offer additional possibilities. In the past, in low-dose CT scans of the head and neck, subject to the decline in image quality after reduction in radiation dose, academic inquiry has always focused on the examination of sinus CT with a simple disease spectrum and low dependence on image quality. With the increasing research on DLIR algorithms, a few frontier studies have focused on experimental research. Romke Rozema [22] used six fresh frozen human cadaver head specimens to quantitatively assess the image quality of advanced modeled iterative reconstruction (ADMIRE) and the PixelShine (PS) deep learning algorithm, concluding that both the ADMIRE and PS algorithms significantly improved image quality after substantial radiation-dose reduction. Our study confirms that the DLIR technique achieves excellent image quality in low-dose pediatric sinus CT. In the next step, we will verify the application of DLIR in other head and neck CT examinations, such as orbital and temporal bone examinations, and relevant studies are in progress.

Although this article briefly discussed our preliminary experience in applying the DLIR algorithm to low-dose CT scans of the paranasal sinuses of children, several limitations of the approach remain. We compared subjective and objective image quality of the DLIR algorithm with that of FBP and ASiR-V but did not conduct a statistical analysis of the lesion detection rates and diagnostic efficacies. Furthermore, the number of patients was relatively small. However, based on the current preliminary research results and combined with the high natural contrast of paranasal sinus CT and the relatively simple disease spectrum of paranasal sinus lesions in children, we have reason to believe that the DLIR algorithm can provide better diagnostic images of low-dose paranasal sinus CT scans in children and increase the feasibility of conducting ultralow-dose scanning in the future. Based on the current study, we will further reduce the tube voltage to 80 kVp and adjust the SmartmA to 35–60 in the next study to achieve ultralow-dose scanning. In the next research step, the diagnostic efficiency of ultralow-dose scanning will also be discussed.

In summary, DLIR had obvious advantages in overall image quality, showing that the DLIR algorithm can maintain high resolution, improve image texture, and reduce image distortion. These advantages cannot be achieved with ASiR-V or other hybrid IR algorithms. Compared to similar studies, our study compares two of the most advanced image reconstruction algorithms: DLIR (not yet commercially available) and ASiR-V (already commercially available). Our study shows that DLIR technology is better than ASiR-V.

## Data Availability

The datasets used and/or analyzed during the current study available from the corresponding author on reasonable request.
